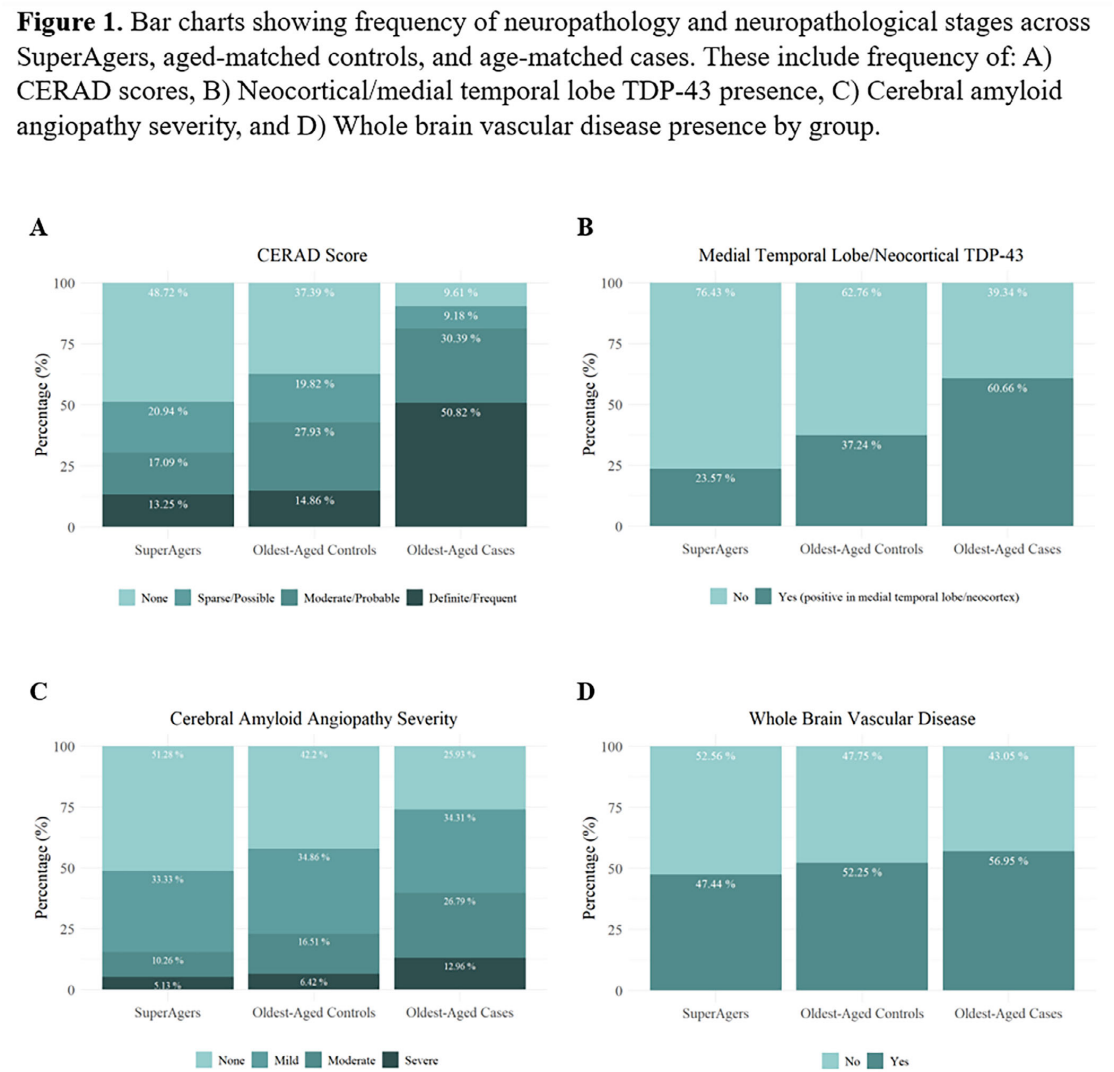# Alzheimer's Disease and concomitant pathologies in SuperAgers

**DOI:** 10.1002/alz70855_106764

**Published:** 2025-12-24

**Authors:** Alaina Durant, Shubhabrata Mukherjee, Michael L. Lee, Seo‐Eun Choi, Phoebe Scollard, Brandon Klinedinst, Emily H. Trittschuh, Jesse Mez, Lindsay A. Farrer, Carlos Cruchaga, Gary W Beecham, Adam C. Naj, Li‐San Wang, Walter W. Kukull, C. Dirk Keene, Andrew J. Saykin, Michael L Cuccaro, Brian W Kunkle, Pedro R Mena, Margaret Pericak‐Vance, Eden R. Martin, David A. A. Bennett, Lisa L. Barnes, Julie A Schneider, William S Bush, Jonathan L Haines, Richard Mayeux, Badri N. Vardarajan, Thomas J. Montine, Paul M. Thompson, Paul K Crane, Logan Dumitrescu, Derek B. Archer, Timothy J. Hohman, Leslie S. Gaynor, Alzheimer's Disease Genetics Consortium (ADGC); The Alzheimer's Disease Sequencing Project (ADSP)

**Affiliations:** ^1^ Vanderbilt University Medical Center, Nashville, TN, USA; ^2^ Department of Medicine, University of Washington, Seattle, WA, USA; ^3^ VA Puget Sound Health Care System, Seattle Division, Seattle, WA, USA; ^4^ Department of Psychiatry and Behavioral Sciences, University of Washington School of Medicine, Seattle, WA, USA; ^5^ Department of Neurology, Boston University Chobanian & Avedisian School of Medicine, Boston, MA, USA; ^6^ Biomedical Genetics, Department of Medicine, Boston University Medical School, Boston, MA, USA; ^7^ Department of Biostatistics, Boston University School of Public Health, Boston, MA, USA; ^8^ NeuroGenomics and Informatics Center, Washington University School of Medicine, St. Louis, MO, USA; ^9^ Washington University School of Medicine, St. Louis, MO, USA; ^10^ Department of Biostatistics and Data Science, Wake Forest School University of Medicine, Winston‐Salem, NC, USA; ^11^ Department of Biostatistics, Epidemiology, and Informatics, and the Penn Neurodegeneration Genomics Center, Department of Pathology and Laboratory Medicine, University of Pennsylvania Perelman School of Medicine, Philadelphia, PA, USA; ^12^ Penn Neurodegeneration Genomics Center, University of Pennsylvania Perelman School of Medicine, Philadelphia, PA, USA; ^13^ Penn Neurodegeneration Genomics Center, Department of Pathology and Laboratory Medicine, University of Pennsylvania Perelman School of Medicine, Philadelphia, PA, USA; ^14^ Department of Epidemiology, School of Public Health, University of Washington, Seattle, WA, USA; ^15^ Department of Laboratory Medicine and Pathology, University of Washington, Seattle, WA, USA; ^16^ Department of Medical and Molecular Genetics, Indiana University School of Medicine, Indianapolis, IN, USA; ^17^ Department of Radiology and Imaging Sciences, Indiana Alzheimer's Disease Research Center, Center for Neuroimaging, Indiana University School of Medicine, Indianapolis, IN, USA; ^18^ John P. Hussman Institute for Human Genomics, University of Miami Miller School of Medicine, Miami, FL, USA; ^19^ Rush Alzheimer's Disease Center, Rush University Medical Center, Chicago, IL, USA; ^20^ Cleveland Institute for Computational Biology, Department of Population and Quantitative Health Sciences, Case Western Reserve University, Cleveland, OH, USA; ^21^ The Taub Institute for Research on Alzheimer's Disease and The Aging Brain, Columbia University Medical Center and The New York Presbyterian Hospital, New York, NY, USA; ^22^ Taub Institute for Research on Alzheimer's Disease and The Aging Brain, Columbia University Medical Center, New York, NY, USA; ^23^ Department of Pathology, Stanford University School of Medicine, Stanford, CA, USA; ^24^ Keck School of Medicine, University of Southern California, Los Angeles, CA, USA; ^25^ Department of General Internal Medicine, University of Washington School of Medicine, Seattle, WA, USA; ^26^ Vanderbilt Genetics Institute, Vanderbilt University Medical Center, Nashville, TN, USA; ^27^ Vanderbilt Memory & Alzheimer's Center, Vanderbilt University Medical Center, Nashville, TN, USA; ^28^ Department of Neurology, Vanderbilt University Medical Center, Nashville, TN, USA; ^29^ Vanderbilt Memory and Alzheimer's Center, Vanderbilt University School of Medicine, Nashville, TN, USA; ^30^ Division of Geriatric Medicine, Department of Medicine, Vanderbilt University Medical Center, Nashville, TN, USA

## Abstract

**Background:**

“SuperAgers” are oldest‐old adults (ages 80+) with memory performance resembling adults in their 50s to mid‐60s. This study assessed presence and levels of Alzheimer's disease (AD) neuropathologic change (ADNC) and concomitant neuropathologies in SuperAgers, AD cases, and oldest‐old controls using three national cohorts.

**Method:**

Harmonized, longitudinal memory, executive function, and language scores and cross‐sectional neuropathology data in Non‐Hispanic White participants were gathered from the ADSP Phenotype Harmonization Consortium (ACT, ROSMAPMARS, and NACC). SuperAgers (*N* = 240) included individuals ages 80+ with a memory score equal to or exceeding individuals ages 50‐64 and language and executive function domain scores within normal limits who remain cognitively normal until death if longitudinal datapoints are available. Analyses also included age‐matched Alzheimer's disease (AD) cases (*N* = 1,181) and cognitively normal controls (*N* = 225). We performed binary logistic regression analyses comparing presence and degree of Alzheimer's Disease neuropathologic change (ADNC), neocortical/medial temporal lobe TDP‐43, hippocampal sclerosis, alpha‐synucleinopathy, cerebrovascular disease, and cerebral amyloid angiopathy (CAA) of SuperAgers and their counterparts, adjusting for sex, education, and age at death. We adjusted for multiple comparisons using the Benjamini‐Hochberg procedure.

**Result:**

Compared to AD dementia cases, SuperAgers had significantly less ADNC and concomitant neuropathologies. SuperAgers, AD dementia cases, and controls had similar presence of microinfarcts, lacunes, and whole brain vascular disease (*p* > 0.05). Compared to age‐matched controls, SuperAgers had lower likelihood and level of neuritic plaques, CAA, and neocortical/medial temporal lobe TDP‐43 (Figure 1).

**Conclusion:**

Across three national cohorts, SuperAgers with optimal memory performance had fewer neuritic plaques, CAA, and neocortical/medial temporal lobe TDP‐43 compared to AD dementia cases and other oldest‐old adults with typical memory performance. In contrast, despite their optimal memory performance, SuperAgers had similar levels of cerebrovascular pathology to AD cases and controls.